# Down-Regulation of miR-126 Is Associated with Colorectal Cancer Cells Proliferation, Migration and Invasion by Targeting IRS-1 via the AKT and ERK1/2 Signaling Pathways

**DOI:** 10.1371/journal.pone.0081203

**Published:** 2013-11-29

**Authors:** Yu Zhou, Xiao Feng, Ya-ling Liu, Shi-cai Ye, Hao Wang, Wen-kai Tan, Ting Tian, Yu-mei Qiu, He-sheng Luo

**Affiliations:** 1 Department of Gastroenterology, Renmin Hospital of Wuhan University, Wuhan, Hubei Province, China; 2 Department of Gastroenterology, The Affiliated Hospital of Guangdong Medical College, Zhanjiang, Guangdong Province, China; University of Saarland Medical School, Germany

## Abstract

**Background:**

Colorectal carcinoma (CRC) is one of the leading causes of cancer-related mortality worldwide. MicroRNAs (miRNAs, miRs) play important roles in carcinogenesis. MiR-126 has been shown to be down-regulated in CRC. In this study, we identified the potential effects of miR-126 on some important biological properties of CRC cells and clarified the regulation of insulin receptor substrate 1 (IRS-1) and its possible signaling pathway by miR-126.

**Methods:**

The effect of miR-126 on IRS-1, AKT, and ERK1/2 expression was assessed in the CRC cell lines HT-29 and HCT-116 with a miR-126 mimic or inhibitor to increase or decrease miR-126 expression. Furthermore, the roles of miR-126 in regulation of the biological properties of CRC cells were analyzed with miR-126 mimic or inhibitor-transfected cells. The 3′-untranslated region (3′-UTR) of IRS-1 regulated by miR-126 was analyzed by using a dual-luciferase reporter assay.

**Results:**

We found that IRS-1 is the functional downstream target of miR-126 by directly targeting the 3′-UTR of IRS-1. Endogenous miR-126 and exogenous miR-126 mimic inhibited IRS-1 expression. Furthermore, gain-of-function or loss-of-function studies showed that over-expression of miR-126 down-regulated IRS-1, suppressed AKT and ERK1/2 activation, CRC cells proliferation, migration, invasion, and caused cell cycle arrest, but had no effect on cell apoptosis. Knockdown of miR-126 promoted these processes in HCT-116 cells and promoted AKT and ERK1/2 activation by up-regulating the expression of the IRS-1 protein.

**Conclusions:**

MiR-126 may play roles in regulation of the biological behavior of CRC cells, at least in part, by targeting IRS-1 via AKT and ERK1/2 signaling pathways.

## Introduction

Colorectal cancer (CRC) is one of the most common human gastrointestinal malignancies in the world with a yearly increasing incidence and mortality rate [1], [2]. It is the fourth leading cause of cancer-related death in both men and women in China [3]. The pathogenesis of CRC is not yet fully understood. It is currently proposed that colorectal carcinogenesis involves multi-step molecular processes with activation of oncogenes, mutation of mismatch repair genes or inactivation of tumor suppressor genes, which affect the proliferation, migration, invasion, apoptosis, or other aspects of cancer cells. In addition to gene activation and inactivation, increasing evidences suggest that microRNAs (miRNAs, miRs) may play roles in the development of CRC [4].

Mature miRNAs are a class of small, non-coding RNA molecules with a length of 20–25 nucleotides. They usually interact with the miRNA-recognition elements in the 3′-untranslated region (3′-UTR) of target mRNAs, regulate mRNA degradation, or repress their translation as important post-transcriptional regulators. MiRNAs have been proven to play critical roles in many biological processes such as cell differentiation, proliferation, apoptosis, inflammatory and immune responses [5], [6]. Increasing evidence has shown that miRNAs are critically involved in tumorigenesis. Depending on the cellular context and target genes that they regulate, miRNAs may function as tumor suppressors or oncogenes [7], [8]. MiR-200 and miR-155 could be involved in cancer cell migration and invasion by regulating the epithelial-to-mesenchymal transition or cellular adhesion [9], [10]. Zhang et al. reported an inverse correlation between metastasis-associated in colon cancer-1(MACC1) and miR-143 expression in colon cancer cell lines and demonstrated that the direct inhibition of metastasis-associated in colon cancer-1 mRNA translation was mediated by miR-143 [11]. Over-expression of miR-211 in HCT-116 cells altered p53 pathway-associated regulatory proteins, e.g., MDM2, Bcl-2, Bcl-xL and Bax [12]. Numerous studies found that miR-126 is significantly decreased in multiple cancer types and, thus, may play a role as tumor suppressor. For instance, low miR-126 expression was observed in non-small cell lung cancer and identified as unfavorable prognostic factor in non-small cell lung cancer patients [13]; miR-126 expression was also decreased in human breast cancer, and may play roles in tumorigenesis and growth by regulating the vascular endothelial growth factor/phosphatidylinositol 3-kinase (PI3K)/AKT signaling pathway [14]. The expression of miR-126 in CRC tissues was significantly lower than that in non-tumor tissues, and miR-126 over-expression inhibited the growth of CRC cells [15]. Guo C et al. noted loss of miR-126 expression in colon cancer cell lines when compared to normal human colon epithelia and revealed that miR-126 regulates PI3K signaling partly by targeting p85β [16]. However, the function of miR-126 and its possible signaling pathway in CRC has not been fully elucidated.

Insulin receptor substrate-1 (IRS-1) is a family member of insulin receptor substrates, which were firstly characterized as typical cytosolic adaptor proteins both in insulin receptor (IR) and insulin-like growth factor I receptor (IGF1R) signaling. Recent studies established that IRS-1 also plays roles in promoting mitosis and apoptosis resistance, malignant transformation and proliferation [17]. Chang et al. [18] found that IRS-1 was over-expressed in various types of solid tumors, including breast cancers, leiomyomas, Wilms' tumors, rhabdomyosarcomas, liposarcomas, leiomyosarcomas and adrenal cortical carcinomas. Moreover, IRS-1 is associated with CRC [19] and up-regulated in cancer cell lines [20]. Bioinformatics has shown that the 3′-UTR of IRS-1 contains a putative binding site for miR-126. However, the regulation of miR-126 in CRC and its association with IRS-1 has not been reported yet.

In this study, we aimed to characterize the roles of miR-126 and its possible signaling pathway in the pathogenesis of CRC cells. In gain-of-function studies, we found that over-expression of miR-126 down-regulated IRS-1 expression, suppressed AKT and ERK1/2 activation, CRC cells proliferation, migration, invasion, and resulted in cell cycle arrest, but had no effect on cell apoptosis. Knockdown of miR-126 promoted these processes in CRC cells and up-regulated the expression of IRS-1 protein. By using luciferase-reporter gene constructs, we identified IRS-1 as functional downstream target of miR-126.

## Materials and Methods

### Cell culture

The CRC cell lines HT-29, HCT-116, SW480 and SW620 were purchased from the cell bank of the tumor hospital of the Chinese Academy of Medical Sciences/Biological Detection Center (Beijing, China). All cells were maintained in RPMI-1640 (Gibco, Carlsbad, CA, USA) containing 10% fetal bovine serum (Gibco), 100 IU/mL penicillin, and 100 µg/mL streptomycin at 37°C in a 5% CO_2_ incubator.

### miRNA mimic or inhibitor transfection

All cells were seeded into 6-well, 12-well, 24-well, or 96-well plates at 90% confluence and kept in an incubator at 37°C and 5% CO_2_ overnight. MiR-126 mimic, miR-126 negative control mimic (NC mimic), miR-126 inhibitor and miR-126 negative control inhibitor (NC inhibitor) were purchased from Ribobio (RiboBio, Guangzhou, China).Varying amounts of miR-126 mimic or NC mimic (RiBoBio) were transfected into HT-29 cells, whereas miR-126 inhibitor or NC inhibitor (RiBoBio) were transfected into HCT-116 cells using Lipofectamine 2000 (Invitrogen, Carlsbad, CA). The transfected cells were incubated at 37°C in a 5% CO_2_ incubator for 24 or 48 h. Total cellular RNA and protein were harvested separately and stored at −80°C until use.

### Identification of potential downstream targets of miR-126

To predict potential targets of miR-126, three in silico analysis programs were used for microRNA target prediction, i.e., MicroCosm Targets (http://www.ebi.ac.uk/enright-srv/microcosm/htdocs/targets/v5/), Targetscan (http://www.targetscan.org/), and PicTar (http://pictar.mdc-berlin.de/). The 3′-UTR of IRS-1 mRNA (RefSeq NM_005544) has a putative miRNA-126 binding site.

### IRS-1 3′-UTR wild type and mutant constructs

In order to test the effects of miR-126 on the expression of IRS-1, we created dual-luciferase reporter constructs. The 3′-UTR of IRS-1 mRNA containing the putative miR-126 binding sequence were synthesized by Sangon Biotech (Shanghai, China). The fragments corresponding to 1-298 nucleotides of the 3′-UTR of IRS-1 mRNA (298 bp) were then subcloned into *Xho*I and *Not*I sites downstream of the Renilla luciferase in the psiCheck-2 vector (Promega). To generate a construct containing the miR-126 binding site mutant, we substituted two nucleotides corresponding to the 5′-seeding region of the miR-126 binding site on the wild type fragment.

Wild type and mutant constructs were designated as psi-IRS-1 and psi-mutIRS-1, respectively. The sequences of the constructs were verified by DNA sequencing. The constructs were transformed into DH5α cells and the plasmid DNA was purified using TIANpure MidiPrep kit (Tiangen Biotech, Beijing, China).

### Construct transfection and luciferase reporter assay

To determine the specific effect of miR-126 on the 3′-UTR of IRS-1, the miR-126 mimic and the psi-IRS-1 luciferase construct (500 ng/well) were co-transfected into HT-29 cells using Lipofectamine 2000. Renilla and Firefly luciferase levels were measured at 48 h post-transfection using the Dual Luciferase Reporter Assay System (Promega Corporation, Madison, WI). Experiments were performed in quadruplicate wells and data represented the mean of three independent experiments.

### Isolation of Total RNA

Cultured cells were rapidly transferred into 1.0 mL of TRIzol reagent (Invitrogen) and total RNA was extracted according to the manufacturer's instructions and stored at −80°C until use.

### Real-time quantitative reverse transcriptase-polymerase chain reaction (qRT-PCR)

The One Step PrimeScript® miRNA cDNA Synthesis Kit and DRR036A (Takara Bio, Inc., Tokyo, Japan) were used to reverse-transcribe the miRNA and mRNA, respectively. For qRT-PCR on transfected cells, 500 ng of total RNA was converted to cDNA. Then, 2 µL of cDNA from each sample was amplified by real-time fluorescent qPCR, using SYBR® Premix Ex Taq™ II (Perfect Real Time) (Takara Bio, Inc.). The expression of miR-126 in each group was calculated relative to that of U6B, a ubiquitously expressed small nuclear RNA used as internal control. Expression of beta-actin was used as normalization control in mRNA qPCR. For miRNA qPCR, the reverse primer was the universal qPCR primer for miRNA (Takara Bio, Inc.). Forward miRNA and mRNA primers were synthesized by Sangon Biotech, and the respective sequences were listed in Table 1. A comparative threshold cycle method was used to compare each condition with the respective controls. The reactions were performed on a LightCycler® (Roche Diagnostics, Basel, Switzerland). The PCR conditions were 30 s at 95°C, followed by 40 cycles of 5 s at 95°C and 20 s at 60°C. The 2^−△Ct^ (2^−[(Ct of gene) − (Ct of U6)]^) method was used for analysis.

**Table 1:** Primers used for quantitative reverse transcription-polymerase chain reaction in this study.

Name	Direction	Primer (5′-3′)
For microRNA qPCR		
Universal qPCR primer	Reverse	The One Step PrimeScript® miRNA cDNA Synthesis Kit (Takara)
miR-126	Forward	TCGTACCGTGAGTAATAATGCG
U6 snRNA	Forward	CTCGCTTCGGCAGCACA
For mRNA qPCR		
IRS-1	Forward	AGTCCTAACCGCAACCAGAGT
	Reverse	CCTCAGCCACACATTCTCAA
Beta-actin	Forward	GGCGGCAACACCATGTACCCT
	Reverse	AGGGGCCGGACTCGTCATACT

### Western blot

Western blot was performed according to the standard protocol. Briefly, 20 µg protein per sample were separated by sodium dodecyl sulfate-polyacrylamide gel electrophoresis and then transferred to polyvinylidene difluoride membranes. The membranes were probed with rabbit anti-human IRS-1 primary antibody (1∶1000 dilution; CST), rabbit anti-human p-AKT primary antibody (1∶500 dilution; CST), rabbit anti-human total-AKT primary antibody (1∶500 dilution; CST), rabbit anti-human p-ERK1/2 primary antibody (1∶500 dilution; CST), or rabbit anti-human total-ERK1/2 primary antibody (1∶500 dilution; CST) overnight at 4°C. The membranes were then incubated with horseradish peroxidase-labeled goat anti-rabbit IgG (1∶1000; Beyotime, Jiangsu, China) at room temperature (RT) for 1 h. Glyceraldehyde-3-phosphate dehydrogenase was used as loading control and a mouse anti-human glyceraldehyde-3-phosphate dehydrogenase antibody was used for its detection. The integral of the optical density of the protein bands was measured using Quantity One image analysis software (Bio-Rad, Hercules, CA).

### Immunofluorescence

Immunofluorescence staining was performed according to a standard protocol (Santa Cruz Biotechnology). Briefly, HCT-116 cells on coverslips were fixed in acetone at 4°C for 10 min and blocked with 10% goat serum albumin at RT for 1 h. The cells were incubated with rabbit anti-human IRS-1 primary antibody (1∶200) (Santa Cruz Biotechnology) overnight at 4°C. After rinsing in phosphate-buffered saline (PBS) for 3 times, the cells were incubated with secondary polyclonal Cy3-labeled goat anti-rabbit-594 IgG (1∶400) (Zhongshan Goldenbridge Biotechnology, Beijing, China) for 1 h at RT in the dark and then washed with PBS. The anti-IRS-1 antibody was replaced with PBS in the negative control sample. 4′,6-diamidino-2-phenylindole was used to counter-stain the nuclei (5 min at RT). The staining was evaluated and the fluorescence intensity was measured on a Leica converted fluorescence microscope. We quantified the total Cy3 fluorescence intensity (as shown in red) as representation of the protein expression level of IRS-1. The intensity of 4′,6-diamidino-2-phenylindole staining (blue) was used as internal normalization control for adjusting fluorescence signals among different slides.

### Cell cycle analysis

Cell cycle analysis was performed by using Cell Cycle and Apoptosis Analysis Kits (Beyotime). HT-29 cells were seeded at a density of approximately 5×10^5^ cells/well into 12-well plates, cultured for 48 h after transfection as mentioned above, harvested, washed with PBS, and then fixed with 70% ethanol overnight at 4°C. Cells were treated with 20 µg/mL RNase, stained with 20 µg/mL propidium iodide for 30 min at 37°C in the dark, and then analyzed by flow cytometry (FACScan; BD Biosciences, San Diego, CA, USA).

### Apoptosis assay

The measurement of apoptosis was conducted according to the manufacturer's instructions for the annexin V-fluorescein isothiocyanate apoptosis detection kit (Beyotime). At 48 h after transfection, harvested cells were suspended in 500 µL annexin V binding buffer. Then, 5 µL annexin V-fluorescein isothiocyanate and 10 µL propidium iodide were added to the wells and cells were incubated for 5 min in the dark. The samples were analyzed within 1 h after staining by flow cytometry (FACScan; BD Biosciences). Cells were discriminated as viable cells, necrotic cells, and apoptotic cells by using the Cell Quest software (BD Biosciences); then, the percentages of apoptotic cells from each group were compared. Assays were performed in triplicate.

### Cell growth assay

Cell viability was examined using the Cell Counting Kit-8 assay (Dojindo Laboratories, Kumamoto, Japan). HT-29 cells were seeded at a density of 5×10^3^ per well in a 96-well plate and cultured as described above for 48 h after transfection. On completion of incubation, cell viability was determined using Cell Counting Kit-8 according to the manufacturer's instructions. The medium was removed and replaced with 100 µL fresh medium supplemented with the Cell Counting Kit-8 reagent (10 µL) in each well and incubated for 1 h at 37°C. The absorbance (A) of each well was read at 450 nm using an enzyme-linked immunosorbent assay reader (Thermo, USA). The cell viability (% of control) was calculated using the following equation: Survival rate (%)  =  (A_sample_ − A_blank_)/(A_control_ − A_blank_), where A_sample_ was the optical density of miR-126-transfected cells, A_control_ was the optical density of NC-transfected cells, and A_blank_ was the optical density of the wells without cells. All experiments were performed in triplicate.

### In vitro transwell migration and invasion assay

Transwell membranes (polycarbonic membrane, diameter 6.5 mm, pore size 8 µm) (Corning Costar, NewYork, USA) coated without or with Matrigel (BD Biosciences) were used to assay migration or invasion of cells in vitro, respectively. At 48 h after transfection, HT-29 or HCT-116 cells were detached by treatment with 0.25% trypsin-EDTA (Gibco), centrifuged, and resuspended in serum-free medium. Transfected cells (10×10^4^ in 200 µL serum-free medium) were reseeded into the upper chamber. Then, 600 µL medium supplemented with 20% fetal bovine serum was added to the lower chamber as chemoattractant. After 48 h of incubation, non-migrating or non-invading cells on the upper surface of the membrane were removed with a cotton swab. The migrated or invaded cells, which penetrated to the lower surface of the membrane, were fixed with 4% paraformaldehyde and stained with 0.1% crystal violet (Beyotime). The number of cells migrating or invading the membrane was counted from 5 randomly selected visual fields, with an inverted microscope at 200× magnification. Results were obtained from three independent experiments.

### Statistical analysis

Experimental results are expressed as mean values ± standard error. Statistical analyses were performed with Student's t-test for 2 groups using SPSS software, v13.0 (International Business Machines Corporation). *P*<0.05 was considered significant.

## Results

### Expression of miR-126 in CRC cell lines

**Figure 1:**
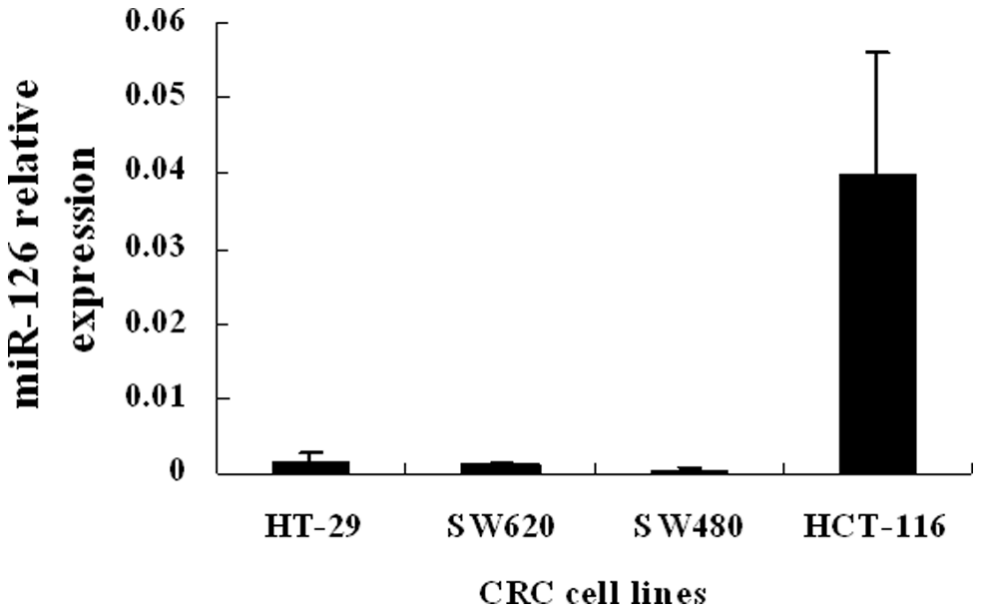
Quantitative reverse transcription-polymerase chain reaction (qRT-PCR) of miR-126 expression in different colorectal cancer (CRC) cell lines. The expression of miR-126 was relatively higher in HCT-116 cells and lower in HT-29, SW620 and SW480 cells among the 4 different CRC cell lines. Data are presented as mean ± SEM of three independent experiments.

We analyzed the expression level of miR-126 in a panel of CRC cell lines with different degrees of differentiation and metastatic ability, including HT-29, HCT-116, SW480 and SW620 cells. We observed that the miR-126 expression was relatively higher in HCT-116 cells than in the other three cell lines. The results shown in Figure 1 suggest that miR-126 expression may be associated with the degree of CRC cells differentiation and metastatic ability. Based on this expression pattern, we chose HT-29 and HCT-116 cells for the following gain-of-function and loss-of-function studies, respectively.

### Selection of potential downstream targets of miR-126

**Figure 2:**
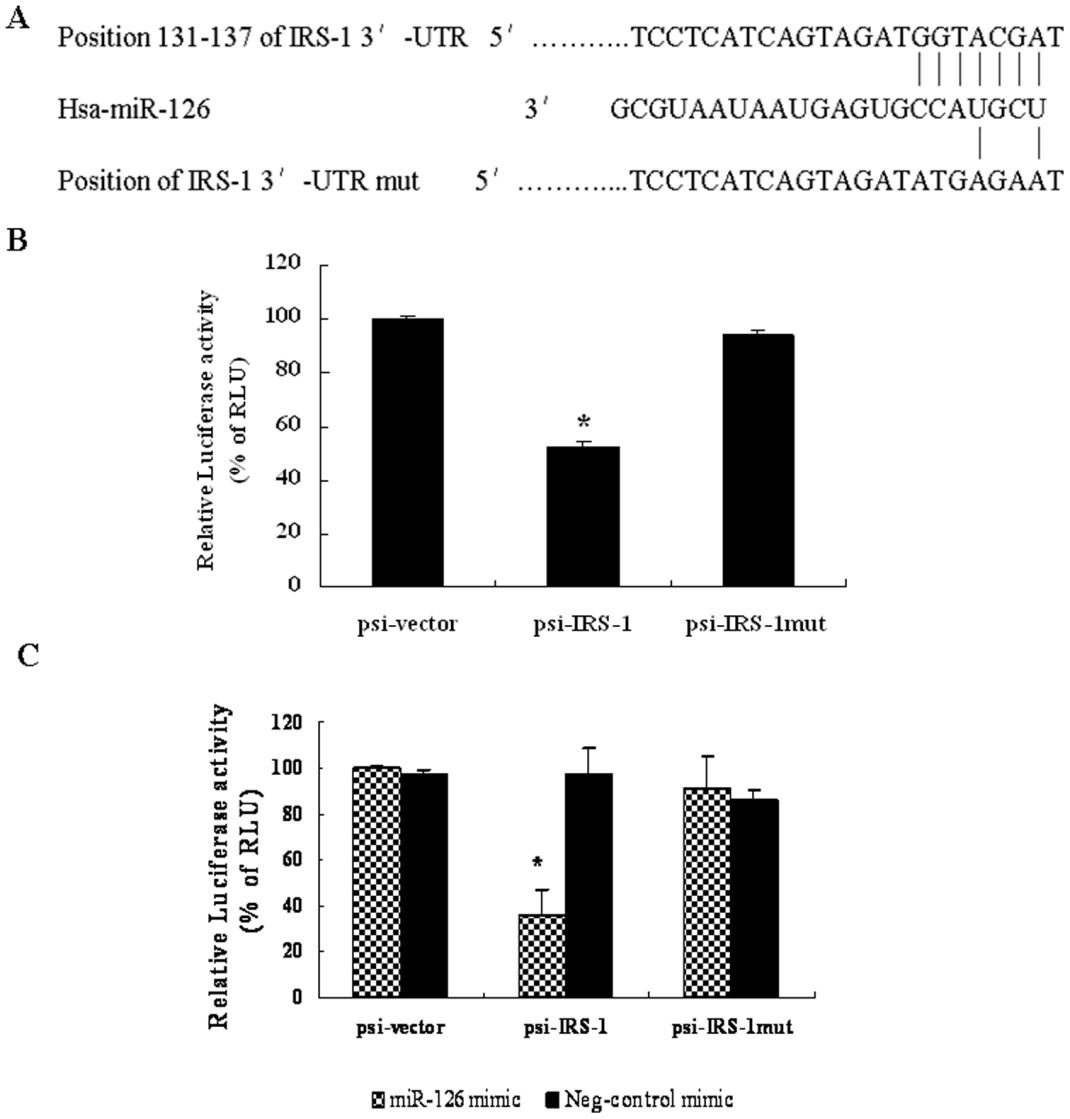
MicroRNA-126 (miR-126) targeting the 3′-untranslated region (UTR) of the insulin receptor substrate 1 (IRS-1). (A) Position of the miR-126 target site in the 3′-UTR of the IRS-1 mRNA predicted by TargetScan. HT-29 cells were transfected with either the original dual luciferase reporter vector (psi-vector) or an IRS-1 3′-UTR construct with the wild type miR-126 binding site (psi-IRS-1) or the mutated miR-126 binding site (psi-IRS-1-mut). Relative Renilla luciferase activity (normalized to Firefly luciferase activity) was measured 24 h post-transfection. (B) Relative Renilla luciferase activity was reduced in cells transfected with the wild type IRS-1 3′-UTR construct (psi-IRS-1) compared to those transfected with the psi-vector (500 ng/well of a 24-well-plate). Relative Renilla luciferase activity was restored in cells transfected with the mutant construct (psi-IRS-1mut). (*P<0.05, compared to that of psi-vector). (C) Co-transfection of HT-29 cells with 50 nM miR-126 mimic and various luciferase reporter constructs (500 ng/well) reduced the relative Renilla luciferase activity. (*P<0.05, compared to that of psi-vector). Data are presented as mean ± SEM of three independent experiments.

MiR-126 was found to be down-regulated in CRC [15]. In order to identify downstream targets of miR-126, we used three miRNA target prediction programs, i.e., MicroCosm Targets, Targetscan, and PicTar, to identify potential targets. Interestingly, IRS-1, which is highly expressed in CRC cells [21], is one of the predicted targets of miR-126. A putative miR-126 binding site that encompasses 6 perfectly matched nucleotides was defined at the 3′-UTR of IRS-1 (Figure 2A).

In an effort to delineate potential downstream targets and further understand the mechanisms of miR-126 down-regulation in the pathogenesis of CRC, we transfected HT-29 cells with an IRS-1 3′-UTR luciferase reporter construct containing a wild type miR-126 putative binding sites (psi-IRS-1) or a mutant construct bearing mutations in miR-126 binding sites (psi-mutIRS-1). The relative luciferase activity of the wild type psi-IRS-1 construct showed 45% reduction when compared to the psi-mutIRS-1 construct (*P*<0.05) (Figure 2B). These results indicate that endogenous miR-126 can regulate IRS-1 expression by directly targeting its 3′-UTR.

To further confirm these findings, the miR-126 mimic was co-transfected with the above luciferase reporter constructs into HT-29 cells. The miR-126 mimic dramatically reduced (>60%) the luciferase activity of the wild type IRS-1 3′-UTR reporter construct psi-IRS-1, whereas the NC mimic had no effect on the luciferase activity in any group (Figure 2C).

However, the miR-126 mimic did not reduce the luciferase activity of the mutant construct psi-mutIRS-1 (Figure 2C), indicating its specific recognition effect. These results further indicate that miR-126 can regulate IRS-1 expression by directly targeting its 3′-UTR.

### Alteration of miR-126 expression changed the IRS-1 protein expression level but not the IRS-1 mRNA level

**Figure 3:**
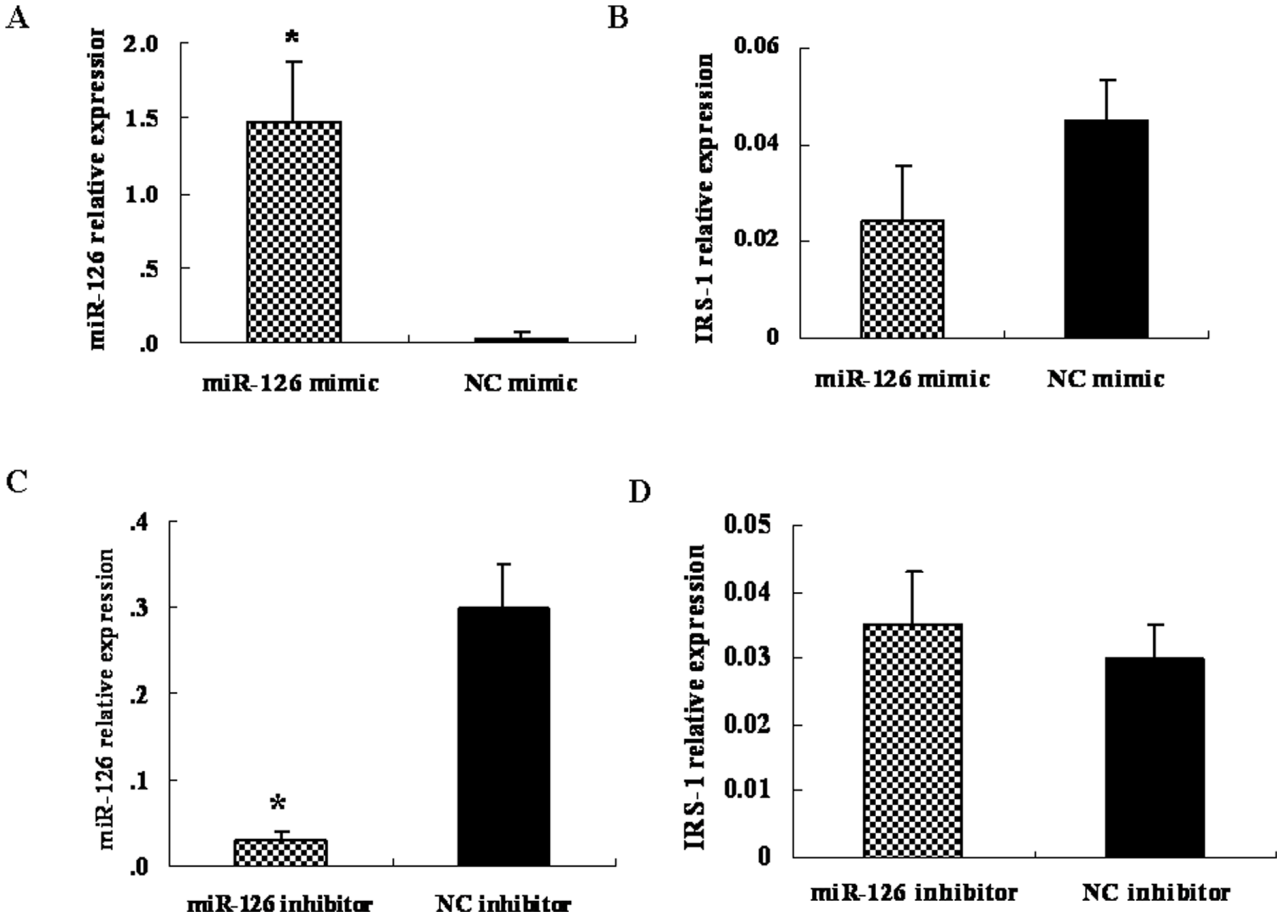
Effects of microRNA 126 (miR-126) mimic or inhibitor on the expression of the insulin receptor substrate 1 (IRS-1) mRNA in human colorectal cancer cell lines. (A, B) HT-29 cells were transfected with 50 nM of miR-126 mimic or negative control mimic for 48 h. Expression of miR-126 and IRS-1 mRNA in the total RNA of these cells was detected by quantitative reverse transcription-polymerase chain reaction analysis. (C, D) HCT-116 cells were transfected with 100 nM of miR-126 inhibitor or negative control inhibitor for 48 h. Expression of miR-126 and IRS-1 mRNA in the total RNA of these cells was detected by qRT-PCR analysis. Data are presented as mean ± SEM of three independent experiments (*P<0.05, compared to that of negative control mimic treatment).

To test whether miR-126 regulates endogenous IRS-1 expression, the miR-126 mimic and inhibitor were transiently transfected into HT-29 and HCT-116 cells, respectively. MiR-126 and IRS-1 mRNA expression levels were assessed. Compared to the NC mimic, transfection with 50 nM of the miR-126 mimic in HT-29 cells led to an approximately 48-fold increase in the miR-126 expression level, as detected by qRT-PCR (Figure 3A). Although there was a decreasing trend in the IRS-1 mRNA expression level in cells transfected with miR-126 mimic, it did not reach statistical significance between the two groups tested (*P*>0.05) (Figure 3B). At the same time, we found that transfection with 100 nM of the miR-126 inhibitor in HCT-116 cells could decrease the mature miR-126 level noticeably (Figure 3C), while the IRS-1 mRNA level remained unchanged (Figure 3D).

**Figure 4:**
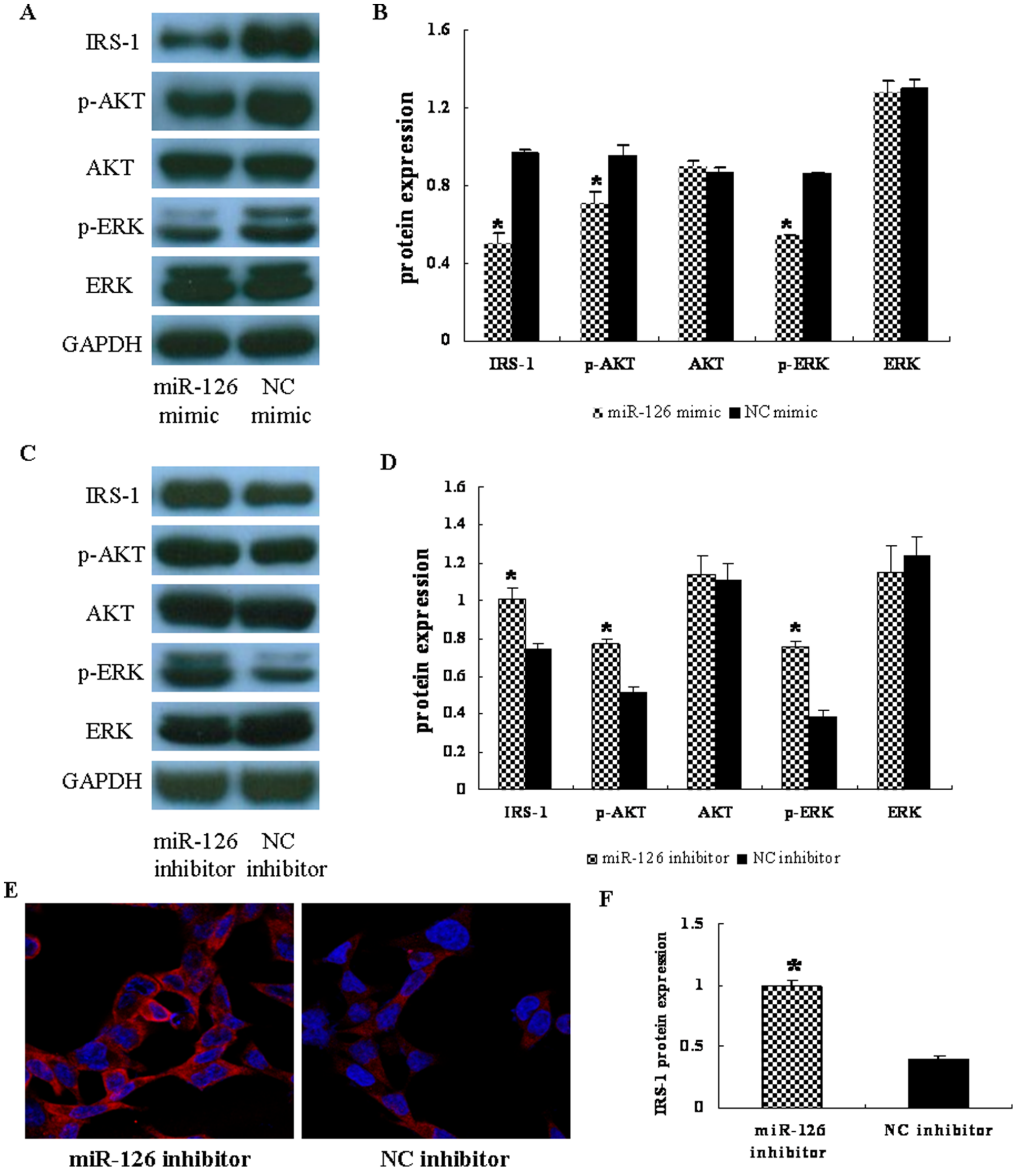
Alteration of microRNA 126 (miR-126) influences AKT and ERK1/2 activation by targeting insulin receptor substrate 1 (IRS-1). (A) HT-29 cells were transfected with miR-126 mimic or negative control (NC) mimic, and total proteins from the cells were used to detect IRS-1, p-AKT, total-AKT, p-ERK1/2, total-ERK1/2, and glyceraldehyde-3-phosphate dehydrogenase (GAPDH) expression by western blotting. (B) Relative protein levels were normalized to those of GAPDH and represented as mean ± SD from three experiments. *, indicates that the expression levels of IRS-1, p-AKT, and p-ERK1/2 were significantly lower in miR-126 mimic transfected cells than that of the negative control (NC) mimic group (*P*<0.05). (C) HCT-116 cells were transfected with miR-126 inhibitor or negative control (NC) inhibitor, and the above proteins were detected by western blotting. (D) Relative protein levels were normalized to those of GAPDH and represented as mean ± SD from three experiments. *, indicates that the expression levels of IRS-1, p-AKT, and p-ERK1/2 were significantly increased in the miR-126 inhibitor group when compared to the NC inhibitor group (*P*<0.05). (E) HCT-116 cells stained for IRS-1 by immunofluorescence. IRS-1 was expressed in the cytoplasm and the levels were significantly increased in miR-126 inhibitor-transfected cells. Red, IRS-1; blue, 4′,6-diamidino-2-phenylindole nuclear staining. Pictures were imaged at 630× magnification on a Leica converted fluorescence microscope. (F) Fluorescence intensity of IRS-1 in each group was then calculated. Data are presented as mean ± SEM of three independent experiments (*P<0.05 compared to that of NC inhibitor).

Next, we determined whether the expression of IRS-1 protein was altered in HT-29 cells transfected with miR-126 mimic or NC mimic and HCT-116 cells transfected with miR-126 inhibitor or NC inhibitor. The increase in miR-126 levels also significantly decreased the IRS-1 protein expression levels as determined by western blot (*P*<0.05) (Figures 4A, B), whereas the mRNA levels remained unchanged (*P*>0.05) (Figure 3B). In contrast, to conduct loss-of-function experiments, 100 nM miR-126 inhibitor was transfected into HCT-116 cells and compared to the NC group. The results showed a decrease in miR-126 expression (Figure 3C) and an increase in IRS-1 protein expression (*P*<0.05) (Figures 4C, D).

### Alteration of miR-126 expression influenced AKT and ERK1/2 activation

To further understand the molecular mechanism of miR-126 in inhibiting tumorigenesis, we found that IRS-1 is a potential novel direct target of miR-126 with a binding site in its 3′-UTR region. IRS-1 can be recruited and phosphorylated by insulin-like growth factor I upon binding to its receptor, insulin-like growth factor I receptor, thus activating downstream signaling pathways such as PI3K/AKT [22]. In this study, we found that the IRS-1 protein expression in HT-29 cells transfected with 50 nM of miR-126 mimic was significantly inhibited (by 47%) as detected by western blot analysis (Figures 4A, B). Consistent with the decrease in the IRS-1 level, over-expression of miR-126 in HT-29 cells also inhibited p-AKT and p-ERK1/2 expression levels (Figures 4A, B). Moreover, transfection of HCT-116 cells with 100 nM of miR-126 inhibitor could up-regulate IRS-1, p-AKT, and p-ERK1/2 protein expression levels, but had no effect on total AKT and ERK1/2 expression levels (Figures 4C, D). These results suggest that miR-126 regulates downstream molecules via targeting IRS-1. In addition, we further performed immunofluorescent staining on HCT-116 cells transfected with miR-126 inhibitor. The staining results showed that the IRS-1 protein was clearly expressed in the cytoplasm of HCT-116 cells (Figure 4E). Its level was markedly increased in the miR-126 inhibitor group when compared to the NC inhibitor group(*P*<0.05) (Figure 4F), which is in agreement with the results obtained by western blotting.

### MiR-126 induced G0/G1 phase arrest in CRC cells

**Figure 5:**
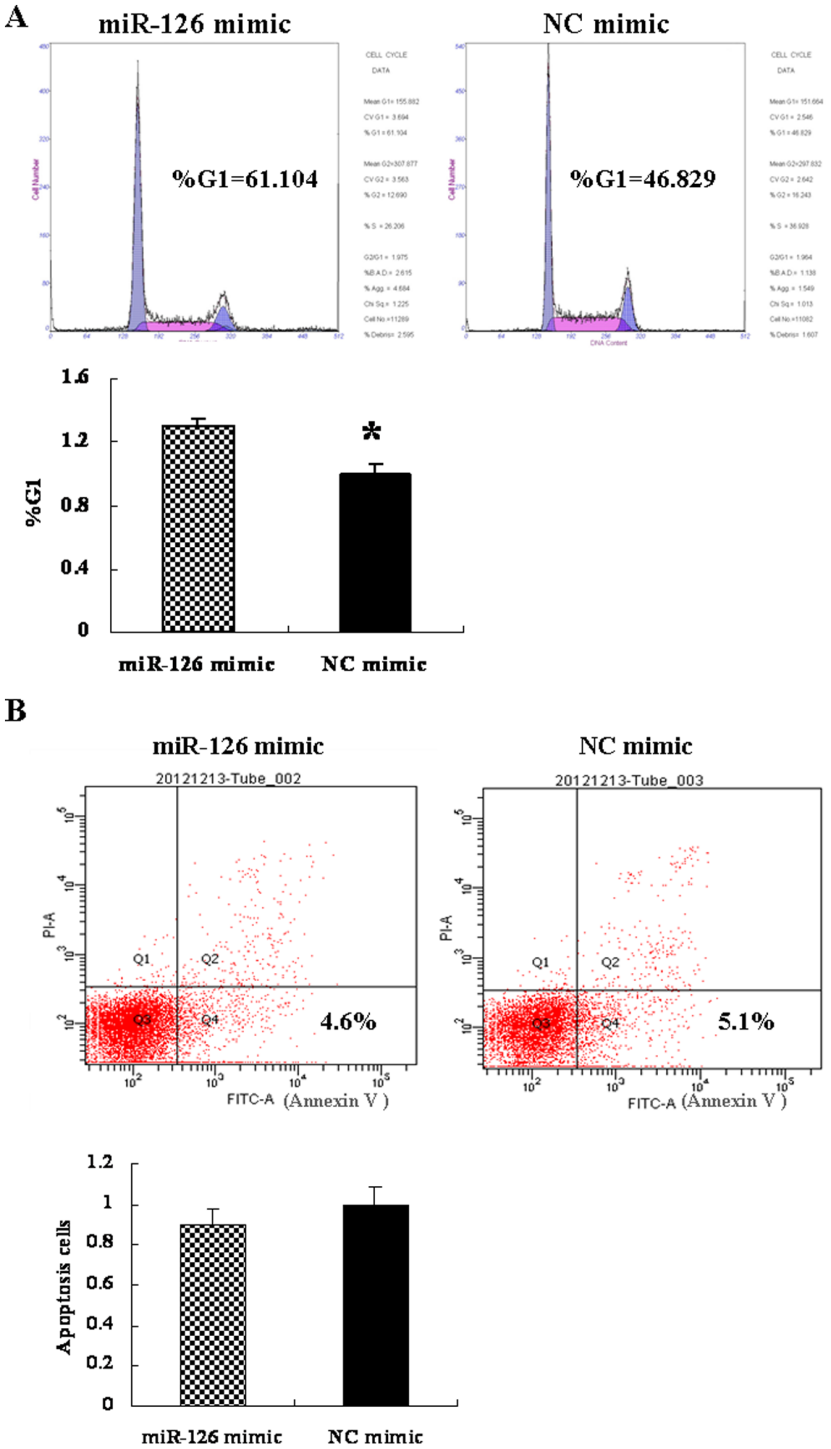
MicroRNA 126 (miR-126) mimic induces G0/G1 phase arrest, but had no effect on cell apoptosis. (A) MiR-126 mimic and NC mimic transfected cells were stained with propidium iodide (PI) and the DNA content was analyzed by flow cytometry. The number of cells in each phase was calculated using ModFit software. The results shown in the bottom graph were representative of three independent experiments (*P<0.05). (B) HT-29 cells were transfected with 50 nM miR-126 mimic or negative control mimic for 48 h. The percentage of apoptotic cells (Annexin V positive) relative to the total measured cell population is shown in the bottom right quadrant of the upper panel. The results were expressed as fold change relative to the corresponding NC mimic (bottom graph). (*P*>0.05, compared to that of NC mimic). Data are presented as mean ± SEM of three independent experiments.

We investigated whether the anti-proliferative activity of miR-126 in HT-29 cells correlated with cell cycle arrest. As demonstrated in Figure 5A, cell cycle analysis revealed that transfection with the miR-126 mimic increased the number of CRC cells in the G0/G1 phase, compared to the NC mimic (*P*<0.05).

### MiR-126 had no effect on apoptosis in CRC cells

To measure the effect of miR-126 on CRC cells apoptosis, apoptosis was measured at 48 h after miR-126 mimic transfection by using flow cytometry. There was no significant difference in the number of annexin V-fluorescein isothiocyanate (+) apoptotic cells in the miR-126 mimic-transfected group compared to the NC mimic-transfected group (Figure 5B, *P*>0.05). These findings indicate that miR-126 might not play an anti-apoptotic role in CRC cells.

### MiR-126 inhibited CRC cells proliferation

**Figure 6:**
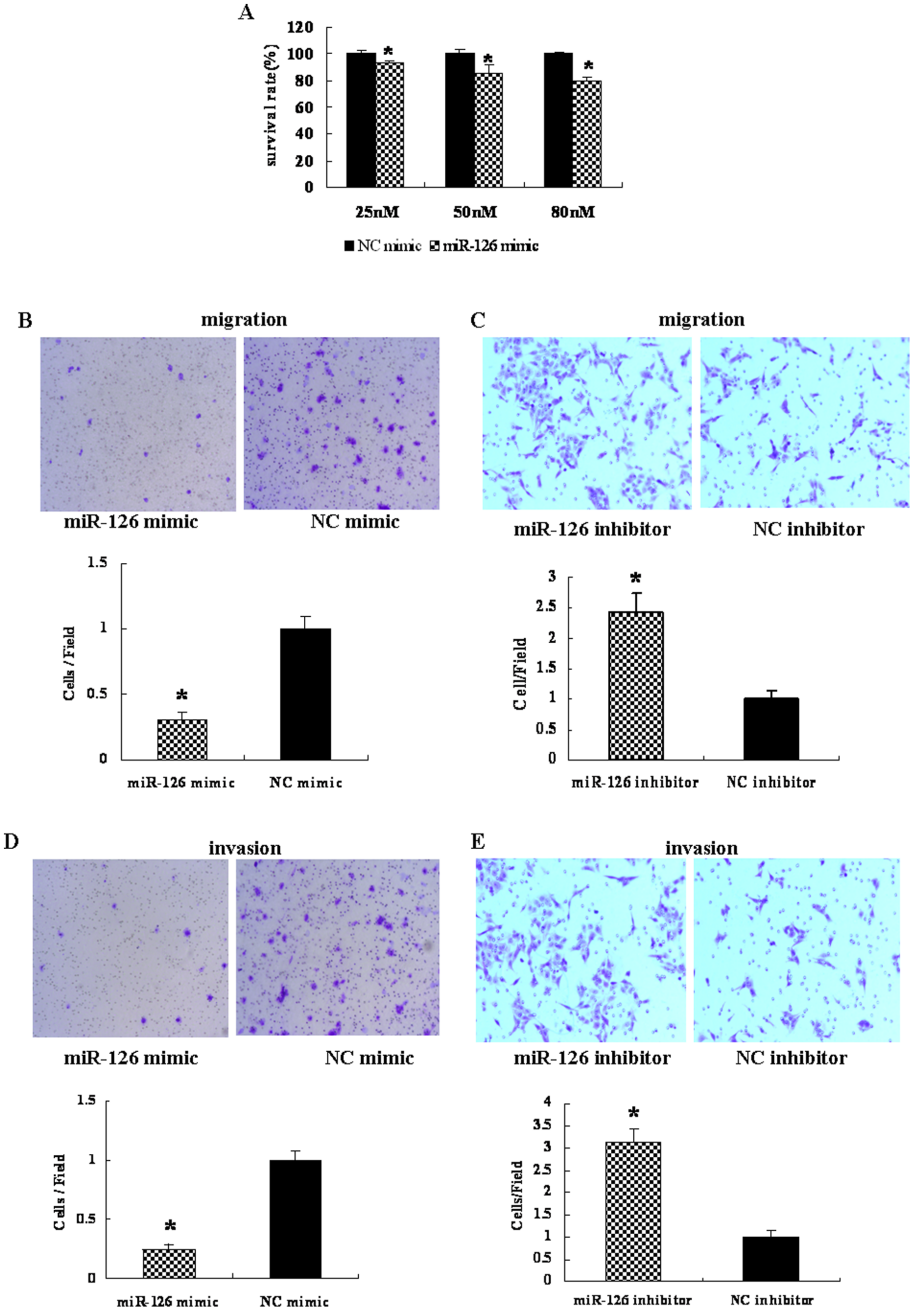
MicroRNA 126 (miR-126) inhibits cell proliferation, migration and invasion of colorectal cancer cells. (A) HT-29 cells were transfected with various amounts of miR-126 mimic or negative control (NC) mimic and cell proliferation was analyzed using Cell Counting Kit-8. The miR-126 mimic showed dose-dependent effects on inhibiting cell proliferation after transfection for 48 h. (*P<0.05, compared to that of NC mimic). Data are presented as mean ± SEM of three independent experiments. HT-29 cells were transfected with 50 nM of miR-126 mimic or NC mimic (B) and HCT-116 cells were transfected with 100 nM of miR-126 inhibitor or NC inhibitor (C), migrated cells stained by 0.1% crystal violet were counted 48 h after transfection (×200). The number of migrated cells per field was obtained from at least three replicate wells and presented as mean ± SD (*P*<0.05) (bottom graph). (D, E) Invaded cells stained by 0.1% crystal violet were counted 48 h after transfection (×200). The number of invaded cells per field was obtained from at least three replicate wells and presented as mean ± SD (*P*<0.05) (bottom graph). *, indicates significant differences between HT-29-miR-126 mimic or HCT-116-miR-126 inhibitor group when compared to each NC group separately (*P*<0.05).

MiR-126 has been reported to be down-regulated in CRC [23], implicating its potential role in the biological properties of CRC cells. To further characterize the functional importance of miR-126 in CRC tumorigenesis, we examined the effect of miR-126 on the proliferation of HT-29 cells using the Cell Counting Kit-8 assay. We observed that over-expression of miR-126 significantly suppressed the proliferation of HT-29 cells at 48 h after transfection (*P*<0.05) (Figure 6A).

### MiR-126 inhibited cell migration and invasion

To test the function of miR-126 in CRC cells, stable cell lines expressing miR-126 (HT-29-miR-126) and negative control (HT-29-NC) were established by Liposome 2000 transduction. Over-expression of miR-126 in HT-29 cells significantly suppressed cell migration (*P*<0.05) (Figure 6B) and cell invasion (*P*<0.05) (Figure 6D), whereas loss of its expression promoted HCT-116 cells migration (*P*<0.05) (Figure 6C) and cells invasion (*P*<0.05) (Figure 6E). These observations suggest that miR-126 plays an important role in inhibiting migration and invasion of CRC cells.

## Discussion

Recent studies have demonstrated that miRNAs play important roles in tumorigenesis by a variety of mechanisms. Firstly, they have been shown to alter tumorous epithelial-mesenchymal transitions and adhesion. For example, over-expression of miR-200 could down-regulate epithelial-to-mesenchymal transition and inhibit tumor migration and invasion [5], [6], [10], [24]. Secondly, they regulate the expression of tumor suppressor genes or oncogenes. MiR-21 is highly expressed in CRC and promotes cancer cell growth by targeting programmed cell death 4 [25]. A similar observation has been made for miR-17-92, which may function as oncogene. On the contrary, under-expressed miRNAs, e.g., let-7, could control cell proliferation and apoptosis as tumor suppressors by regulating oncogenes [26]. Thirdly, miRNAs regulate the expression of metastasis-associated gene in cancers [11]. In addition, miRNAs are involved in DNA methylation and regulate vessel formation, thereby influencing tumor development [27], [28].

Numerous studies have demonstrated that miR-126 is associated with inflammation as well as cancers. Over-expression of miR-126 was found to inhibit placenta-specific 1(Plac1) and further increase the viability of gastric cancer by targeting sex-determining region Y-box 2 (SOX2) as oncogene [29]. On the other hand, miR-126 was shown to function as a tumor suppressor in breast cancer [30], lung cancer [31], [32], and bladder cancer [33], cervical cancer [34] by targeting different genes. MiR-126 was down-regulated in CRC tissues [15]. Increased miR-126 expression could suppress CRC cells proliferation [15]. These findings suggest that miR-126 regulates different molecular targets in different cancer processes. However, the function of miR-126 in CRC carcinogenesis remains unknown.

In this study, we found that the expression of miR-126 was relatively low in HT-29, SW480, SW620 cells and high in HCT-116 cells, indicating that miR-126 may be associated with the biological behavior of CRC cells. Therefore, we chose HT-29 cells for subsequent gain-of-function and HCT-116 cells for loss-of-function studies. In this study, we studied the function and potential mechanisms of miR-126 in regulating the biological behavior of CRC cells and its possible signaling pathway. Our results showed that miR-126 inhibited proliferation, migration, invasion, and resulted in cell cycle arrest of CRC cells, but had no effect on cell apoptosis in vitro. To characterize the potential mechanisms of miR-126-mediated changes in the biological properties of CRC cells, we identified molecular targets of miR-126 through target scan analysis. We found that the 3′-UTR of IRS-1 mRNA possesses a miR-126-targeted sequence.

IRS-1, a docking protein, is highly expressed in many kinds of cancers where it acts as an oncogene, e.g., pancreatic cancer [35] and breast cancer [18]; we found that IRS-1 is mainly expressed in the cytoplasm of the CRC cell line HCT-116. Transgenic mice over-expressing IRS-1 or IRS-2 in the mammary gland showed progressive mammary hyperplasia, tumorigenesis, and metastasis [36]. In addition, over-expression of IRS-1 can target upstream binding factor-1 (UBF-1) and, therefore, affect cell size [37]. These findings indicate that IRS-1 may play major roles on in cell growth, proliferation, and differentiation. We also found that insertion of the wild type of miR-126 binding site in the IRS-1 3′-UTR in a dual-luciferase reporter construct led to a reduction of the reporter gene expression and the mutant construct showed the restoration of the reporter gene expression. Moreover, over-expression of miR-126 induced the inhibition of IRS-1, specifically.

It has been reported that miRNA may play roles in CRC tumorigenesis via different signaling pathways. For example, miR-145 and miR-101 have been shown to be under-expressed and promote Wnt/β-catenin signaling in human colon cancer cells [38], [39]. In addition, miR-145 also inhibited p-ERK expression by targeting PAK4, leading to inhibition of tumor growth [40]. Another study showed that miR-31 could activate the RAS signaling pathway through inhibiting RASA1 translation [41]. Down-regulation of miR-144 leads to poor prognosis of CRC patients via activation of the mTOR signaling pathway [42]. All of these findings indicate that CRC processes are regulated by various miRNAs and signaling pathways. Guo et al. found that miR-126 may act as a tumor suppressor in CRC by down-regulating the p58β subunit through the PI3K signaling pathway [16]. Another study indicated that miR-126 suppresses colon cancer cell proliferation and invasion via inhibition of the RhoA/ROCK signaling pathway [23]. These observations raise the question: Could miR-126 be involved in CRC tumorigenesis through a third pathway?

AKT- and ERK1/2-mediated signaling pathways are complex biological processes. It has been confirmed that IRS-1 transduces extracellular signals into cells through AKT and ERK1/2 signaling pathways via miR-145 in CRC cells [21]. Here, we demonstrated that endogenous miR-126 and exogenous miR-126 mimic can inhibit IRS-1 expression. We also found that down-regulation of miR-126 could promote AKT and ERK1/2 activation by targeting IRS-1.Taken together, our results imply that up-regulation of miR-126 could down-regulate IRS-1, inhibit CRC cells proliferation, migration, invasion, and induce cell cycle arrest, but not in apoptosis of CRC cells, partly through AKT and ERK1/2 signaling pathways. Moreover, the results of our study and previous studies further confirme that miRNA and its target genes are not in a one-to-one correspondence relationship, but instead, one miRNA can regulate several pathways by targeting different mRNAs, and one target mRNA can be regulated by several miRNAs and pathways in the same human tumor.

## Conclusion

In conclusion, our study demonstrated previously unknown biological functions of miR-126 in CRC cells. In addition, IRS-1 was down-regulated at the post-transcriptional level via a binding site of miR-126 in the 3′-UTR of IRS-1 mRNA. These findings suggest that miR-126 is possibly involved in the tumorigenesis of CRC, at least to some extent, by suppression of IRS-1 through AKT and ERK1/2 signaling pathways, which would have important implications for further understanding the signaling mechanisms involved in modulating tumorigenesis.
